# Levels of exposure markers among residents in environmentally vulnerable areas in Korea, the general population in Korea, and Asians in the United States

**DOI:** 10.4178/epih.e2025007

**Published:** 2025-02-25

**Authors:** Kyung-Hwa Choi, Dahee Han, Sang-Yong Eom, Yong Min Cho, Young-Seoub Hong, Woo Jin Kim

**Affiliations:** 1 Department of Preventive Medicine, Dankook University College of Medicine, Cheonan, Korea; 2 Institute of Environmental Health, Seokyeong University, Seoul, Korea; 3 Department of Environmental Chemical Engineering, Seokyeong University, Seoul, Korea; 4 Department of Preventive Medicine, Chungbuk National University, College of Medicine, Cheongju, Korea; 5 Department of Preventive Medicine, Dong-A University College of Medicine, Busan, Korea; 6 Department of Internal Medicine, Kangwon National University, College of Medicine, Chuncheon, Korea

**Keywords:** FROM study, Environmentally vulnerable, Biomarkers

## Abstract

This study compares biomarker levels among environmentally vulnerable residents in Korea, the general Korean population, and Asians in the United States. We selected 953 exposed residents and 204 controls from the Forensic Research via Omics Markers in Environmental Health Vulnerable Areas (FROM) study (2021-2023), 4,239 participants from the fourth Korean National Environmental Health Survey (2018-2020), and 996 Asians from the U.S. National Health and Nutrition Examination Survey (2017-March 2020). The analyzed biomarkers included blood and urinary metals, urinary metabolites of polycyclic aromatic hydrocarbons, nicotine, volatile organic compounds, and serum perfluorocarbon metabolites. The highest median biomarker levels varied by pollution source among older adults. In refineries, blood lead and cadmium (Cd), as well as urinary Cd and 2-hydroxyfluorene, were highest. Abandoned metal mines exhibited the highest blood and urinary mercury, urinary Cd, total arsenic (As), 2-naphthol, and cotinine levels. Coal-fired power plants showed the highest urinary 1- hydroxyphenanthrene levels, while cement factories had the highest urinary As^3+^ levels. Sprawls demonstrated the highest urinary monomethylarsonic acid, 1-hydroxypyrene, and phenylglyoxylic acid levels, and industrial areas recorded the highest levels of trans, trans-muconic acid, benzylmercapturic acid, and 2-methylhippuric acid. In general, biomarker levels were higher among exposed residents in the FROM study than in the general population; however, urinary 2-hydroxyfluorene and As^5+^ levels did not differ significantly. Exposure to pollution sources in environmentally vulnerable areas may elevate biomarker levels in residents.

## GRAPHICAL ABSTRACT

[Fig f3-epih-47-e2025007]

## Key Message

This study examines environmental health risks for vulnerable populations by comparing biomarker levels among exposed residents in Korea, the general Korean population, and Asians in the United States. Biomarker levels were found to be elevated near pollution sources such as refineries, metal mines, and power plants, with variations based on pollutant types. The scientific and epidemiological significance lies in revealing disparities in exposure and potential health effects, thereby contributing to targeted interventions for environmentally vulnerable groups.

## INTRODUCTION

Residents living in environmentally vulnerable areas are exposed to pollution from multiple sources [1], as highlighted by numerous incidents in countries such as Korea and the United States.

Biomarker concentrations in individuals reflect their level of exposure to environmental pollutants [2]. These concentrations are associated with various adverse health effects and serve as early indicators of potential health risks [2]. As pollutants enter the body and are metabolized into different compounds, biomarkers provide a useful measure of exposure.

Each metal has distinct adverse health effects. Lead (Pb) accumulates in the brain, liver, kidneys, and bones [3]. Mercury (Hg) exposure can lead to brain damage, paralysis, incoherent speech, and delirium [4]. The kidneys are particularly vulnerable to heavy metal toxicity. Chronic exposure to cadmium (Cd) may result in osteomalacia and osteoporosis, especially in areas contaminated with Cd [5]. Inorganic arsenic (As) is a known carcinogen linked to skin, bladder, and lung cancers, and it also contributes to diabetes, pulmonary disease, cardiovascular disease, and adverse pregnancy outcomes [6]. Assessing metal exposure typically involves measuring their concentrations in the body. For Pb, only blood levels are measured, while both blood and urine are used for other metals.

Humans can be exposed to volatile organic compounds (VOCs) via inhalation, ingestion, and dermal contact. VOCs exposure may cause irritation of the eyes, nose, throat, lungs, and skin, as well as damage to the liver, kidneys, and central nervous system. It can also lead to increased pulse rate, headaches, and shortness of breath [7]. Urinary metabolites that serve as indicators of VOCs exposure include trans, trans-muconic acid (t,t-MA) for benzene; hippuric acid (HA) and benzylmercapturic acid (BMA) for toluene; phenylglyoxylic acid (PGA) for styrene; and 2-methylhippuric acid (2-MHA) for xylene.

Polycyclic aromatic hydrocarbons (PAHs) levels are influenced by both the duration and route of exposure. Long-term exposure to PAHs can lead to diminished immune function, cataracts, kidney and liver damage (e.g., jaundice), respiratory problems, asthma-like symptoms, and impaired lung function [8]. Urinary metabolites of PAHs include 2-naphthol (a marker for naphthalene), 1-hydroxypyrene (1-OHP) for pyrene, 2-hydroxyfluorene (2-OHFlu) for fluorene, and 1-hydroxyphenanthrene (1-PHEN) for phenanthrene.

The health effects of perfluorocarbons (PFCs) remain uncertain due to limited research. Nevertheless, exposure to PFCs has been associated with adverse birth outcomes, disruptions in thyroid function, and increased cholesterol levels. These exposures are typically assessed by measuring serum concentrations of perfluorooctanoic acid (PFOA), perfluorooctanesulfonic acid (PFOS), perfluorohexanesulfonic acid (PFHxS), perfluorononanoic acid (PFNA), and perfluorodecanoic acid (PFDeA) [9].

Cotinine, a biomarker of tobacco exposure, is associated with adverse pregnancy outcomes, acute lower respiratory infections, reduced lung function, and an increased risk of pulmonary disease, asthma, and cardiovascular disease [10].

Therefore, this study aimed to compare and evaluate biomarker concentrations among environmentally vulnerable residents in Korea, the general Korean population, and Asians in the United States.

## MATERIALS AND METHODS

### Study population and participants

This study included participants from the Forensic Research via Omics Markers in Environmental Health Vulnerable Areas (FROM) study, the Korean National Environmental Health Survey (KoNEHS), and the U.S. National Health and Nutrition Examination Survey (NHANES).

**Figure 1.**
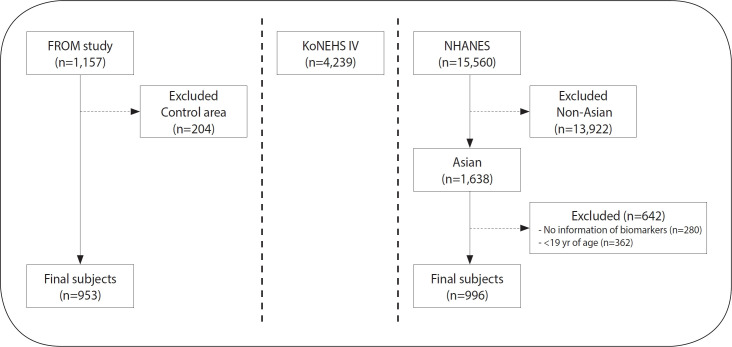
Selection process of participants in the FROM study, KoNEHS IV, and NHANES. FROM study, Forensic Research via Omics Markers in Environmental Health Vulnerable Area Study; KoNEHS IV, the fourth Korean National Environmental Health Survey (2018-2020); NHANES, National Health and Nutrition Examination Survey (2017-Mar 2020).

The FROM study in Korea (2021-2025) examines environmental pollutant characteristics in vulnerable areas by analyzing biological samples to develop markers for assessing health effects and tracking diseases. Survey areas were selected based on their classification as environmentally vulnerable regions, which include sites near abandoned metal mines, smelters, industrial complexes, incinerators, cement factories, and sprawls [11]. Participants were recruited on a voluntary basis after receiving detailed information about the study’s objectives. In total, 1,157 participants were enrolled in the FROM study between 2021 and 2023, comprising 953 from vulnerable areas and 204 from control areas. Details are provided elsewhere [2]. For the current study, we included 953 participants from vulnerable areas and 204 from control areas (Figure 1).

The KoNEHS has been conducted annually since 2009 to monitor chemical exposure in the general Korean population [12]. Each 3-year survey cycle includes 5-6 thousand participants. For this study, we used data from the fourth survey cycle (2018-2020), which included 4,239 adult participants (Figure 1).

The NHANES is a nationally representative, population-based survey that assesses the health and nutritional status of the general United States population. Since 1999, approximately 5,000 participants have been selected annually using a complex, multistage probability sampling design [13]. For this study, we used data collected between 2017 and March 2020. Out of 15,560 participants, we excluded 13,922 non-Asians, 280 participants without biomarker information, and 362 participants aged below 19 years, leaving a total of 996 Asian participants (Figure 1).

### Biomarkers

The analyzed biomarkers comprised blood and urinary metals, urinary metabolites of PAHs, nicotine, VOCs, and serum PFCs. The experimental methods for determining biomarker levels varied among the surveys (Supplementary Material 1). Concentrations below the limit of detection (LOD) were replaced with LOD/sqrt(2), and urinary biomarker levels were normalized using urinary creatinine concentrations.

#### Metals

Both the FROM study and NHANES-Asian analyzed biomarkers for metals, including blood Pb; blood and urinary Hg and Cd; and urinary total As, As^5+^, As^3+^, and monomethylarsonic acid (MMA) levels. The detection rates of metals, except As^3+^, were higher in the FROM study than in the other surveys. Among the surveys, the LODs for metals in blood and urine were the lowest in the FROM study (Supplementary Material 2).

#### PAHs

The FROM study and KoNEHS IV analyzed urinary PAHs metabolites, including 1-OHP, 2-naphthol, 2-OHFlu, and 1-PHEN. In the FROM study, urinary PAHs metabolite detection rates were higher and LODs lower compared to KoNEHS IV (Supplementary Material 2).

#### Nicotine

Urinary cotinine, a nicotine metabolite, was measured in both the FROM study and KoNEHS IV. In the FROM study, the detection rate for urinary cotinine was lower and the LODs were higher compared to KoNEHS IV. NHANES data were not comparable, as only serum cotinine levels were analyzed (Supplementary Material 2).

#### VOCs

For urinary VOCs metabolites, the FROM study measured t,t-MA, BMA, PGA, and 2-MHA. The detection rates in the FROM study were lower than those in the KoNEHS study but higher than in the NHANES-Asian study. In particular, both the LOD and detection rate for urinary 2-MHA were lower in the NHANES-Asian population compared to the FROM study (Supplementary Material 2).

#### PFCs

To assess PFCs exposure, serum levels of PFOA, PFOS, PFHxS, PFNA, and PFDeA were measured. The LODs and detection rates for these PFCs were similar between the FROM study and KoNEHS IV (Supplementary Material 2).

### Covariates

All surveys collected data on age, sex, body mass index (BMI, kg/m²), smoking history, and alcohol intake via questionnaires. These variables were categorized as follows: age (19-<45, 45-<60, and ≥60 years), sex (male, female), BMI (<18.5, 18.5-<23.0, 23.0-<25.0, 25.0-<30.0, ≥30.0 kg/m², and unknown), smoking history (never, past, current, and unknown), and alcohol intake (no, yes, and unknown).

### Statistical analysis

The chi-square test was used to assess differences in general characteristics across surveys. The Kruskal–Wallis test evaluated differences in biomarker distributions among the surveys. Adjusted p-values were computed using log-transformed exposure markers via analysis of variance, with adjustments for age, sex, BMI, smoking status, and alcohol intake. Additionally, the older age group was analyzed separately, as they were considered more suitable for evaluating long-term exposure to pollution.

### Ethics statement

This study was approved by the Institutional Review Board of Dong-A University of Korea (2-1040709-AB-N-01-202105-BR-002-08). Informed consent was obtained from all participants, and the study followed the guidelines of the Declaration of Helsinki for research on human participants.

## RESULTS

Table 1 presents the distribution of general characteristics across surveys. Participants from the FROM study’s exposed areas were predominantly female (65.0%) and were mostly older adults (87.5% aged ≥60 years). Their smoking (7.9%) and alcohol intake (63.4%) rates were lower compared to controls in the FROM study and the general Korean population, but higher than those observed in NHANES-Asian. The obesity rate (45.2%) was higher than that of the controls in the FROM study, similar to the general Korean population, and lower than that of NHANES-Asian.

Table 2 displays the distribution of biomarkers across surveys. Levels of blood Pb, Hg, blood and urinary Cd, urinary total As, MMA, 2-naphthol, 1-PHEN, BMA, and 2-MHA, as well as serum PFDeA, were higher in the FROM study compared to the general population.

Within the FROM study, median biomarker levels varied according to the source of pollution (Table 3). In refineries, the highest levels observed were blood Pb (2.60 μg/dL), blood Cd (2.19 μg/L), and urinary Cd (1.93 μg/g cr). In areas near abandoned metal mines, the highest levels were seen for blood Hg (4.70 μg/L), urinary Hg (0.97 μg/g cr), urinary total As (424.07 μg/g cr), and cotinine (5.34 μg/g cr). Waste incinerators exhibited the highest urinary 2-naphthol levels (4.51 μg/g cr), while coal-fired power plants showed the highest urinary 1-PHEN levels (0.24 μg/g cr). Cement factories had the highest urinary As^3+^ (1.68 μg/g cr), and sprawls demonstrated the highest levels of urinary MMA (45.79 μg/g cr), 1-OHP (0.22 μg/g cr), PGA (303.8 μg/g cr), and 2-MHA (200.47 μg/g cr). Additionally, industrial areas recorded the highest t,t-MA (93.17 μg/g cr) and BMA (10.75 μg/g cr). Notably, the highest urinary 2-OHFlu level (0.32 μg/g cr) was observed in the KoNEHS, and the highest As^5+^ level (0.76 μg/g cr) was found in NHANES-Asian.

**Figure 2.**
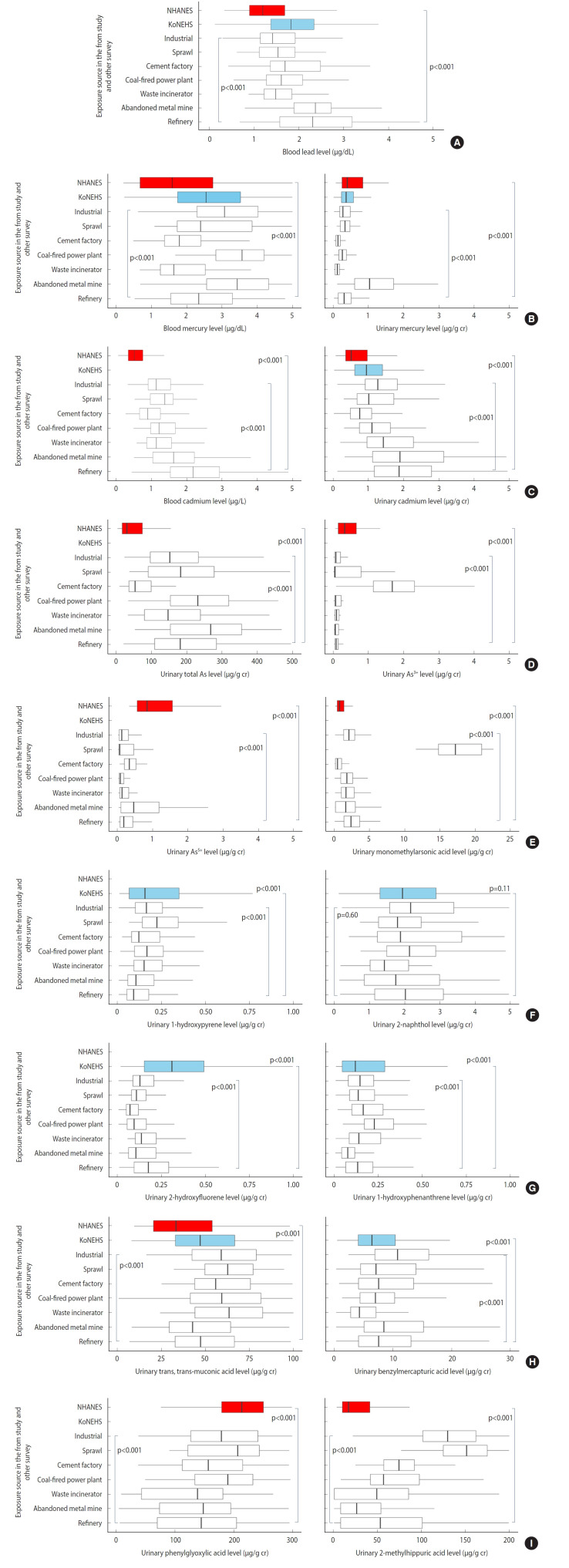
Distribution of exposure biomarkers by exposure source among the exposed groups of the FROM study, KoNEHS IV, and NHANES in older adults. (A-E) Blood and urinary metals; (F, G) Urinary metabolites of PAHs; (H, I) Urinary metabolites of VOCs. The p-value estimated using the Kruskal-Wallis test. FROM study, Forensic Research via Omics Markers in Environmental Health Vulnerable Area Study; KoNEHS IV, the fourth Korean National Environmental Health Survey (2018-2020); NHANES, National Health and Nutrition Examination Survey (2017-Mar 2020); PAHs, polycyclic aromatic hydrocarbons; VOCs, volatile organic compounds; As, arsenic.

Figure 2 illustrates the distribution of biomarkers among older adults by exposure source in the FROM study and the other surveys. The results are similar to those presented in Table 3, although older adults generally exhibited higher biomarker values compared to the overall participant group. Detailed results are provided in Supplementary Materials 3 and 4.

## DISCUSSION

This study found that biomarker concentrations in residents of environmentally vulnerable areas in Korea were generally higher than those observed in the general Korean population and Asians in the United States. This was particularly evident for blood Pb and Hg, blood and urinary Cd, urinary total As, As^5+^, MMA, 2-naphthol, 1-PHEN, BMA, 2-MHA, and serum PFDeA. Among older adults, the highest levels of metals and PAHs were found in refineries; metals and VOCs in abandoned metal mines; PAHs in waste incinerators; PAHs and VOCs in coal-fired power plants; inorganic As in cement factories; and MMA, PAHs, and VOCs in sprawls, while industrial areas exhibited the highest VOCs levels.

In the FROM study, blood and urinary heavy metal levels were higher than those observed in the general population of Korea and Asians in the United States. Notably, approximately 40% of FROM study participants resided near smelters and abandoned mines, regions known for elevated metal concentrations compared to other exposed areas.

Furthermore, the FROM study observed the highest VOCs levels in areas near abandoned metal mines and coal-fired power plants. This observation is consistent with previous research, which has shown that coal-fired power plants emit VOCs [14], and that atmospheric benzene and heavy metal concentrations around power plants are elevated [15]. Although few studies have examined abandoned mine areas, the FROM study detected high VOCs metabolite concentrations among residents in these regions. It is worth noting that mining activities can release biogenic VOCs, which play important roles in ecological interactions [16].

Inorganic As exhibited the highest levels in cement factory areas, in line with previous findings. A study in the United States reported that residents near cement factories had elevated inorganic As concentrations in their bodies [17]. In Korea, cement factories utilize domestic and industrial waste—including discarded tires, plastic, vinyl, textiles, wood, and oil—as fuel for their kilns instead of fossil fuels. This practice can lead to the emission of PAHs, dioxins, and heavy metals into the atmosphere [18]. Therefore, residents living near cement plants may be exposed to a range of pollutants, including carcinogens.

Urinary 1-OHP and 2-OHFlu levels were higher in KoNEHS than in the FROM study. In the FROM study, urinary 1-OHP levels were low in smelter and abandoned metal mine areas, where incineration did not occur. A Mexican study reported similar findings, with no cases exceeding the 1-OHP reference level in smelter areas, whereas cases above the reference level were observed in regions with heavy traffic, brick manufacturing, and landfills [19]. Additionally, no significant difference in urinary 1-OHP levels was observed between the control area and either Hoboken, which hosts a lead smelter, or Wilrijk, where a waste incinerator/printing plant is located [20]. However, these results remain controversial. In contrast, repeated measurements of urinary PAHs metabolites in residents near an aluminum plant in Quebec, Canada, revealed higher levels in the exposed group compared to controls [21]. This discrepancy may be due to several factors. First, increased fine dust concentrations could result in similarly high PAHs levels in the general population, obscuring differences with vulnerable areas. Second, the short half-life of PAHs in the human body may limit their ability to accurately reflect exposure levels. Third, differences in measurement periods may play a role; we compared general population data collected before coronavirus disease 2019 (COVID-19) with vulnerable population data collected during COVID-19. A systematic review of COVID-19’s environmental impacts reported a significantly greater reduction in carbon dioxide emissions in the aviation sector compared to the power and industry sectors, which are related to the exposure sources in this study [22]. The review also noted an increase in medical waste from incineration, another relevant exposure source [22]. Therefore, a repeated-measures study design might yield more accurate evaluations. It should be noted that the data in this study were derived from cross-sectional surveys (FROM, KoNEHS, and NHANES), which have inherent limitations.

The As^5+^ concentration was higher in the NHANES-Asian data compared to the FROM study. This discrepancy may stem from differences in LOD and detection rates for As^5+^. In NHANES-Asian, the detection rate for As^5+^ was 6.6%, with the LOD corresponding to the 93.4th percentile and the median corresponding to the 96.7th percentile among undetected samples—approximately the top 3.3% overall. In contrast, the FROM study had a detection rate of 49.1%, which makes it appropriate to compare the 98.5th percentile among detected samples (representing the top 3%) with NHANES-Asian data. In the FROM study, the 98.5th percentile (3.25 μg/g cr) exceeds the median (0.76 μg/g cr) observed in the NHANES-Asian group.

This study had some limitations. First, in the FROM study, blood and urine sampling and chemical analyses were conducted over a long period with various seasons involved changes in analytical instruments, and encompassed unique circumstances such as the COVID-19 pandemic. Nonetheless, both internal and external quality controls were implemented to ensure accuracy and precision. Second, as a cross-sectional study, it is challenging to ascertain whether the observed biomarker concentrations were directly affected by environmental pollutants. To address this, we included an older age group (aged ≥60 years) and adjusted for health behaviors such as smoking, alcohol consumption, and BMI when analyzing differences related to residential environments. Third, although the total sample size was adequate, the number of samples decreased when subdividing by pollution source.

Despite these limitations, this study aimed to assess exposure to various pollutants by analyzing multiple biomarkers in vulnerable areas. Moreover, we identified and listed the pollutants to which residents in these areas are predominantly exposed.

## Figures and Tables

**Figure f3-epih-47-e2025007:**
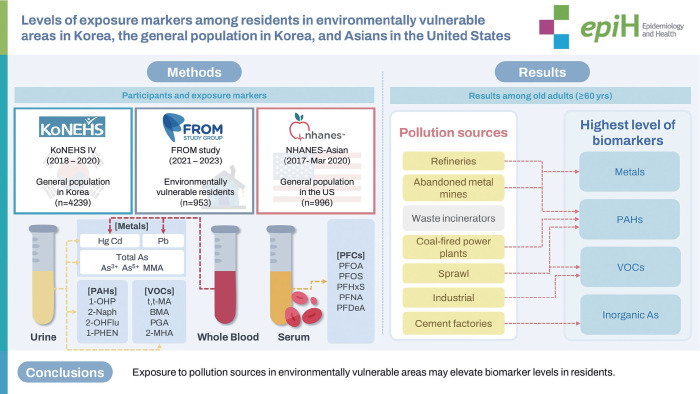


**Table 1.** Distribution of general characteristics in the FROM study, KoNEHS IV, and NHANES

Characteristics		FROM study		KoNEHS IV (2018-2020)	Asians in the NHANES (2017-Mar 2020)	p-value^1^
		Exposed	Control			
All		953	204	4,239	996	
Age (yr)	Median (Min-Max)	71 (19-95)	68 (40-93)	54 (19-82)	48 (19-80)	
	19-<45	19 (2.0)	7 (3.4)	1,372 (32.4)	419 (42.1)	<0.001
	45-<60	100 (10.5)	31 (15.2)	1,301 (30.7)	318 (31.9)	
	≥60	834 (87.5)	171 (83.8)	1,566 (36.9)	259 (26.0)	
Sex	Male	334 (35.0)	77 (37.7)	1,889 (44.6)	457 (45.9)	<0.001
	Female	619 (65.0)	127 (62.3)	2,350 (55.4)	539 (54.1)	
Body mass index (kg/m^2^)	Median (Min-Max)	24.5 (11.3-40.7)	23.9 (17.5-30.5)	24.5 (13.8-49.5)	25.3 (14.6-60.6)	
	<18.5	25 (2.6)	5 (2.5)	96 (2.3)	22 (2.2)	<0.001
	18.5-<23.0	271 (28.4)	81 (39.7)	1,244 (29.3)	249 (25.0)	
	23.0-<25.0	225 (23.6)	50 (24.5)	1,018 (24.0)	188 (18.9)	
	25.0-<30.0	354 (37.1)	58 (28.4)	1,547 (36.5)	373 (37.4)	
	≥30.0	77 (8.1)	9 (4.4)	334 (7.9)	153 (15.4)	
	Unknown	1 (0.1)	6 (2.9)	0	11 (1.1)	
Cigarette smoking history	Never	683 (71.7)	141 (69.1)	2,731 (64.4)	796 (79.9)	<0.001
	Past	195 (20.5)	44 (21.6)	817 (19.3)	130 (13.1)	
	Current	75 (7.9)	19 (9.3)	691 (16.3)	70 (7.0)	
	Unknown	0	5 (2.5)	0	0	
Alcohol consumption	No	349 (36.6)	85 (41.7)	846 (20.0)	128 (12.9)	<0.001
	Yes	604 (63.4)	119 (58.3)	3,393 (80.0)	514 (51.6)	
	Unknown	0	5 (2.5)	0	354 (35.5)	

Values are presented as number (%). FROM study, Forensic Research via Omics Markers in Environmental Health Vulnerable Area Study; KoNEHS IV, the fourth Korean National Environmental Health Survey (2018-2020); NHANES, National Health and Nutrition Examination Survey (2017-Mar 2020); Min, minimum; Max, maximum.

1 Using the chi-square test.

**Table 2.** Distribution of blood and urinary level of biomarkers among the exposed groups in the FROM study, KoNEHS IV, and NHANES

Exposure markers		Unit	Exposed in the FROM study (n=953)	KoNEHS IV (2018-2020) (n=4,239)	Asians in the NHANES (2017-Mar 2020) (n=996)	p-value^1^
Metals (blood)	Lead	μg/dL	1.82 (1.32-2.57)	1.61 (1.18-2.11)	1.14 (0.77-1.68)	<0.001
	Mercury	μg/L	3.34 (2.05-4.99)	2.99 (1.96-4.77)	1.60 (0.50-3.59)	<0.001
	Cadmium	μg/L	1.35 (0.94-1.98)	-	0.42 (0.25-0.66)	<0.001
Metals (urine)	Mercury	μg/L	0.19 (0.09-0.40)	0.27 (0.16-0.47)	0.22 (0.09-0.49)	<0.001
		μg/g cr	0.28 (0.15-0.54)	0.35 (0.22-0.56)	0.34 (0.18-0.63)	<0.001
	Cadmium	μg/L	0.95 (0.48-1.72)	0.48 (0.23-0.89)	0.24 (0.11-0.51)	<0.001
		μg/g cr	1.31 (0.83-2.20)	0.64 (0.32-1.09)	0.37 (0.18-0.68)	<0.001
	Total As	μg/L	130.34 (62.82-254.10)	-	13.05 (5.48-36.35)	<0.001
		μg/g cr	193.01 (99.54-348.98)	-	17.31 (7.73-38.07)	<0.001
	As^5+^	μg/L	0.10 (0.04-0.33)	-	0.56 (0.56-0.56)	<0.001
		μg/g cr	0.15 (0.06-0.46)	-	0.76 (0.48-1.32)	<0.001
	As^3+^	μg/L	0.04 (0.03-0.34)	-	0.08 (0.08-0.86)	<0.001
		μg/g cr	0.07 (0.04-0.40)	-	0.35 (0.14-0.74)	<0.001
	Monomethylarsonic acid	μg/L	1.23 (0.50-2.54)	-	0.48 (0.14-1.12)	<0.001
		μg/g cr	1.87 (0.80-3.16)	-	0.67 (0.33-1.20)	<0.001
Polycyclic aromatic hydrocarbons metabolites (urine)	1-Hydroxypyrene	μg/L	0.10 (0.05-0.22)	0.16 (0.03-0.33)	-	<0.001
		μg/g cr	0.14 (0.08-0.27)	0.18 (0.07-0.40)	-	0.010
	2-Naphthol	μg/L	2.48 (1.03-6.37)	2.51 (1.16-6.29)	-	0.170
		μg/g cr	3.55 (1.75-8.19)	2.99 (1.65-6.62)	-	0.001
	2-Hydroxyfluorene	μg/L	0.10 (0.04-0.21)	0.25 (0.12-0.50)	-	<0.001
		μg/g cr	0.13 (0.08-0.27)	0.32 (0.17-0.56)	-	<0.001
	1-Hydroxyphenanthrene	μg/L	0.09 (0.05-0.18)	0.10 (0.03-0.21)	-	0.880
		μg/g cr	0.14 (0.08-0.24)	0.12 (0.04-0.27)	-	0.001
Nicotine metabolite	Cotinine	μg/L	1.57 (0.77-5.17)	2.36 (0.97-8.37)	-	<0.001
		μg/g cr	2.41 (1.05-7.73)	2.43 (1.19-11.12)	-	<0.001
Volatile organic compounds metabolites (urine)	Trans, trans-muconic acid	μg/L	47.26 (27.15-87.63)	49.71 (27.19-101.64)	28.00 (13.38-60.33)	<0.001
		μg/g cr	69.67 (44.24-120.48)	62.37 (37.92-106.34)	35.35 (22.39-67.04)	<0.001
	Benzylmercapturic acid	μg/L	6.00 (3.19-12.27)	4.30 (2.30-8.30)	-	<0.001
		μg/g cr	9.05 (4.84-16.26)	5.09 (3.13-8.76)	-	<0.001
	Phenylglyoxylic acid	μg/L	123.63 (70.07-223.12)	-	171.00 (92.35-259.00)	<0.001
		μg/g cr	197.25 (118.93-292.29)	-	210.68 (164.06-277.66)	<0.001
	2-Methylhippuric acid	μg/L	75.37 (32.35-161.97)	-	11.30 (5.34-24.25)	<0.001
		μg/g cr	107.89 (47.00-214.90)	-	15.39 (9.91-26.51)	<0.001
Perfluorocarbons (serum)	Perfluorooctanoic acid	μg/L	6.49 (4.37-8.71)	7.15 (4.87-10.20)	-	<0.001
	Perfluorooctanesulfonic acid	μg/L	14.34 (10.19-20.07)	17.11 (11.19-25.58)	-	<0.001
	Perfluorohexanesulfonic acid	μg/L	3.08 (2.15-4.18)	4.33 (2.74-6.95)	-	<0.001
	Perfluorononanoic acid	μg/L	2.11 (1.48-2.63)	2.46 (1.58-3.66)	-	<0.001
	Perfluorodecanoic acid	μg/L	1.23 (0.87-1.70)	1.04 (0.70-1.50)	-	<0.001

Values are presented as median (interquartile range). FROM study, Forensic Research via Omics Markers in Environmental Health Vulnerable Area Study; KoNEHS IV, the fourth Korean National Environmental Health Survey (2018-2020); NHANES, National Health and Nutrition Examination Survey (2017-Mar 2020); As, arsenic.

1 Using log transformed exposure markers and analysis of variance adjusted for age, sex, body mass index, smoking status, and alcohol intake.

**Table 3.** Distribution of exposure biomarkers by exposure sources among the exposed groups in the FROM study, Korea

Exposure markers		Unit	Refineries (n=243)	Abandoned metal mines (n=142)	Waste incinerator (n=55)	Coal-fired power plants (n=113)	Cement factories (n=92)	Sprawl (n=68)	Industrial (n=240)	p-value^1^
Metals (blood)	Lead	μg/dL	2.60 (1.76-3.97)	2.37 (1.89-2.74)	1.49 (1.22-1.87)	1.63 (1.31-2.15)	1.74 (1.37-2.49)	1.54 (1.11-1.93)	1.43 (1.13-1.88)	<0.001
	Mercury	μg/L	2.68 (1.76-3.90)	4.70 (3.09-6.95)	1.73 (1.25-2.68)	4.56 (3.40-6.80)	1.85 (1.38-2.69)	3.41 (1.85-5.40)	3.70 (2.57-5.01)	<0.001
	Cadmium	μg/L	2.19 (1.55-3.12)	1.61 (0.96-2.45)	1.14 (0.86-1.54)	1.20 (0.90-1.59)	0.91 (0.68-1.26)	1.29 (0.91-1.61)	1.15 (0.90-1.55)	<0.001
Metals (urine)	Mercury	μg/L	0.16 (0.09-0.31)	0.85 (0.42-1.49)	0.07 (0.04-0.15)	0.20 (0.11-0.31)	0.12 (0.06-0.20)	0.14 (0.09-0.28)	0.20 (0.09-0.34)	<0.001
		μg/g cr	0.30 (0.14-0.50)	0.97 (0.58-1.71)	0.11 (0.06-0.20)	0.25 (0.13-0.39)	0.13 (0.07-0.20)	0.32 (0.19-0.47)	0.26 (0.18-0.47)	<0.001
	Cadmium	μg/L	1.20 (0.61-2.04)	1.46 (0.91-2.45)	0.80 (0.42-1.28)	0.86 (0.52-1.45)	0.72 (0.31-1.31)	0.52 (0.30-0.92)	0.81 (0.37-1.36)	<0.001
		μg/g cr	1.93 (1.16-3.00)	1.83 (1.07-3.13)	1.38 (0.95-2.27)	1.03 (0.70-1.50)	0.75 (0.48-1.15)	1.00 (0.67-1.62)	1.23 (0.82-1.75)	<0.001
	Total As	μg/L	122.05 (67.53-242.45)	294.81 (207.57-594.76)	85.12 (50.29-145.61)	182.36 (102.18-333.17)	49.57 (24.10-104.49)	115.40 (65.00-166.90)	109.64 (51.00-214.00)	<0.001
		μg/g cr	208.42 (118.45-338.86)	424.07 (232.72-788.61)	144.98 (83.30-255.44)	237.90 (145.30-380.10)	52.16 (34.06-97.10)	211.42 (127.47-362.01)	165.17 (97.26-254.30)	<0.001
	As^5+^	μg/L	0.08 (0.04-0.32)	0.40 (0.06-1.09)	0.04 (0.04-0.27)	0.04 (0.04-0.16)	0.25 (0.13-0.43)	0.02 (0.02-0.22)	0.03 (0.03-0.23)	<0.001
		μg/g cr	0.18 (0.06-0.48)	0.43 (0.08-1.19)	0.12 (0.06-0.35)	0.08 (0.04-0.18)	0.33 (0.19-0.52)	0.06 (0.03-0.36)	0.11 (0.04-0.31)	<0.001
	As^3+^	μg/L	0.04 (0.04-0.04)	0.03 (0.03-0.03)	0.04 (0.04-0.04)	0.04 (0.04-0.20)	1.39 (0.86-2.64)	0.01 (0.01-0.46)	0.03 (0.03-0.03)	<0.001
		μg/g cr	0.07 (0.04-0.15)	0.05 (0.03-0.11)	0.09 (0.05-0.20)	0.06 (0.04-0.24)	1.68 (1.13-2.31)	0.05 (0.03-0.78)	0.06 (0.03-0.18)	<0.001
	Monomethylarsonic acid	μg/L	1.46 (0.67-2.95)	0.98 (0.06-2.71)	1.11 (0.54-1.78)	1.19 (0.53-2.06)	0.27 (0.10-1.15)	23.63 (14.97-44.75)	1.19 (0.76-2.03)	<0.001
		μg/g cr	2.23 (1.27-3.46)	1.20 (0.09-2.43)	1.61 (1.09-2.65)	1.69 (0.79-2.54)	0.40 (0.12-0.96)	45.79 (29.72-80.39)	1.90 (1.19-2.71)	<0.001
Polycyclic aromatic hydrocarbons metabolites (urine)	1-Hydroxypyrene	μg/L	0.07 (0.03-0.15)	0.09 (0.05-0.21)	0.12 (0.05-0.21)	0.11 (0.07-0.23)	0.11 (0.06-0.29)	0.12 (0.05-0.23)	0.12 (0.06-0.24)	<0.001
		μg/g cr	0.10 (0.06-0.20)	0.11 (0.06-0.24)	0.16 (0.09-0.26)	0.17 (0.10-0.26)	0.12 (0.08-0.26)	0.22 (0.14-0.38)	0.17 (0.10-0.35)	<0.001
	2-Naphthol	μg/L	2.48 (1.00-5.11)	3.32 (1.05-8.96)	2.35 (0.59-8.34)	3.11 (1.41-8.81)	2.84 (1.18-5.52)	1.41 (0.74-4.23)	2.18 (1.05-5.80)	0.030
		μg/g cr	3.77 (1.85-8.68)	3.88 (1.52-9.47)	4.51 (1.33-10.65)	3.14 (1.89-8.50)	3.34 (1.51-6.02)	2.90 (1.66-7.59)	3.51 (1.89-7.61)	0.680
	2-Hydroxyfluorene	μg/L	0.12 (0.06-0.24)	0.12 (0.05-0.23)	0.12 (0.05-0.44)	0.09 (0.04-0.18)	0.07 (0.03-0.14)	0.06 (0.03-0.13)	0.10 (0.04-0.20)	<0.001
		μg/g cr	0.20 (0.10-0.36)	0.12 (0.07-0.28)	0.17 (0.10-0.67)	0.10 (0.06-0.19)	0.07 (0.05-0.13)	0.11 (0.08-0.19)	0.14 (0.09-0.23)	<0.001
	1-Hydroxyphenanthrene	μg/L	0.08 (0.04-0.16)	0.06 (0.03-0.11)	0.10 (0.05-0.16)	0.20 (0.12-0.35)	0.14 (0.07-0.32)	0.08 (0.03-0.13)	0.09 (0.05-0.16)	<0.001
		μg/g cr	0.14 (0.07-0.23)	0.08 (0.04-0.12)	0.14 (0.08-0.24)	0.24 (0.17-0.35)	0.16 (0.10-0.28)	0.13 (0.09-0.23)	0.15 (0.08-0.24)	<0.001
Nicotine metabolite	Cotinine	μg/L	3.37 (1.01-7.27)	4.60 (2.02-9.70)	1.16 (0.64-64.43)	1.21 (0.59-2.01)	1.48 (0.40-3.82)	1.04 (0.25-3.23)	1.09 (0.67-1.88)	<0.001
		μg/g cr	5.21 (2.01-14.48)	5.34 (2.30-14.37)	1.96 (1.01-126.03)	1.44 (0.83-2.68)	1.43 (0.53-4.86)	1.79 (0.60-8.00)	1.80 (0.89-3.36)	<0.001
Volatile organic compounds metabolites (urine)	trans, trans-Muconic acid	μg/L	36.64 (19.98-69.90)	45.84 (24.49-85.21)	43.20 (25.67-91.99)	65.53 (36.56-116.72)	53.98 (40.91-92.73)	38.04 (25.99-58.43)	57.47 (30.69-101.47)	<0.001
		μg/g cr	57.43 (36.72-96.93)	54.07 (33.79-84.16)	83.10 (56.37-121.47)	84.34 (54.65-124.43)	65.47 (45.34-95.57)	76.69 (55.35-116.57)	93.17 (55.78-153.74)	<0.001
	Benzylmercapturic acid	μg/L	5.22 (2.77-10.54)	8.91 (4.82-19.58)	2.44 (1.31-5.77)	6.00 (3.56-10.62)	6.74 (3.24-14.49)	4.64 (2.37-13.44)	6.05 (3.90-11.73)	<0.001
		μg/g cr	8.30 (4.19-14.09)	10.14 (5.39-22.78)	3.96 (2.71-7.28)	7.80 (4.05-12.29)	7.89 (4.32-14.15)	9.20 (5.25-22.41)	10.75 (7.15-17.47)	<0.001
	Phenylglyoxylic acid	μg/L	91.05 (39.81-177.84)	115.36 (60.12-232.87)	62.41 (28.76-133.04)	206.83 (121.74-328.29)	143.61 (97.01-248.14)	150.03 (95.40-206.37)	122.98 (81.95-210.30)	<0.001
		μg/g cr	165.99 (80.09-244.64)	162.09 (94.70-231.82)	154.16 (50.61-232.57)	238.28 (172.06-343.86)	175.36 (123.00-263.80)	303.80 (206.30-393.10)	222.11 (146.58-309.36)	<0.001
	2-Methylhippuric acid	μg/L	49.77 (13.86-123.50)	32.34 (7.88-117.55)	48.05 (20.45-150.44)	55.34 (36.50-123.01)	88.13 (39.69-147.05)	115.24 (64.03-186.51)	115.49 (74.20-239.46)	<0.001
		μg/g cr	72.83 (21.82-149.36)	35.07 (7.88-84.47)	56.54 (37.80-230.96)	72.61 (45.52-123.83)	81.26 (62.86-140.60)	200.47 (148.94-300.58)	197.25 (132.60-298.76)	<0.001

Values are presented as median (interquartile range). FROM study, Forensic Research via Omics Markers in Environmental Health Vulnerable Area Study; As, arsenic.

1 Using the Kruskal-Wallis test.

## Supplementary materials

Supplementary Material 1. Experimental methods of biomarkers in the FROM study, KoNEHS IV, and NHANES

Supplementary Material 2. Limit of detection and detection rate of specimens by exposure markers in the FROM study, KoNEHS IV, and NHANES

Supplementary Material 3. Distribution of exposure biomarkers among different exposure sources in the FROM study among participants older than 60 years of age

Supplementary Material 4. Distribution of exposure biomarkers by different exposure sources and control groups in the FROM study, KoNEHS IV, and NHANES-Asian among participants older than 60 years of age
