# Supplementary material for: Levels of exposure markers among residents in environmentally vulnerable areas in Korea, the general population in Korea, and Asians in the United States
Source: Epidemiol Health. 2025 Feb 25;47:e2025007. doi: 10.4178/epih.e2025007 (PMC12062851; doi:10.4178/epih.e2025007)
Supplement: Supplementary Material 1. — Experimental methods of biomarkers in the FROM study, KoNEHS IV, and NHANES [file epih-47-e2025007-Supplementary-1.docx]

**Levels of exposure markers among residents in environmentally vulnerable areas in Korea, the general population in Korea, and Asians in the USA**

**Supplementary Materials**

**Supplementary Material 1. Experimental methods of biomarkers in the FROM study, KoNEHS IV, and NHANES**

| Exposure markers | | FROM study | KoNEHS IV (2018-2020) | NHANES (2017-Mar 2020) |
| --- | --- | --- | --- | --- |
| Metals (Blood) | Lead | ICP-MS, Inductively Coupled Plasma-Mass Spectrometer | GF-AAS, Graphite Furnace-Atomic Absorption Spectrometer | Blood Multi-Element ICP-DRC-MS, Inductively Coupled Plasma-Mass Spectrometry |
|  | Mercury | Gold amalgamation direct mercury analyzer | |  |
|  | Cadmium | ICP-MS | - |  |
| Metals (Urine) | Mercury | Gold amalgamation direct mercury analyzer | | Urine Multi-Element ICP-DRC-MS |
|  | Cadmium | ICP-MS | GF-AAS |  |
|  | Total arsenic | ICP-MS | - | ICP-MS |
|  | As5+ | HPLC-ICP-MS, High Performance Liquid Chromatography Inductively Coupled Plasma-Mass Spectrometry | - | HPLC-ICP-DRC-MS, High Performance Liquid Chromatography Inductively Coupled Plasma Dynamic Reaction Cell Mass Spectrometry |
|  | As3+ |  |  |  |
|  | Monomethylarsonic acid (MMA) |  |  |  |
| Polycyclic aromatic hydrocarbons metabolites (Urine) | 1-Hydroxypyrene | GC-MS, Gas Chromatography-Mass Spectrometry | | - |
|  | 2-Naphthol |  |  |  |
|  | 2-Hydroxyfluorene |  |  |  |
|  | 1-Hydroxyphenanthrene |  |  |  |
| Nicotine metabolite (Urine) | Cotinine | LC-MS/MS, liquid chromatography -Mass Spectrometry | GC-MS | - |
| Volatile organic compounds metabolites (Urine) | trans, trans-Muconic acid | LC-MS/MS | HPLC-MS/MS, high performance liquid chromatography - electrospray ionization-mass spectrometer | UPLC-ESI-MS/MS, Ultra Performance Liquid Chromatography with Electro Spray Tandem Mass Spectrometry |
|  | Benzylmercapturic acid |  |  | - |
|  | Phenylglyoxylic acid |  |  | UPLC-ESI-MS/MS |
|  | 2-Methylhippuric acid |  |  |  |
| Perfluorocarbons (Serum) | Perfluorooctanoic acid (PFOA) | HPLC-MS/MS | | - |
|  | Perfluorooctanesulfonic acid (PFOS) |  |  |  |
|  | Perfluorohexanesulfonic acid (PFHxS) |  |  |  |
|  | Perfluorononanoic acid (PFNA) |  |  |  |
|  | Perfluorodecanoic acid (PFDeA) |  |  |  |

FROM study, Forensic Research via Omics Markers in Environmental Health Vulnerable Area Study; KoNEHS IV, The Fourth Korean National Environmental Health Survey (2018-2020); NHANES, National Health and Nutrition Examination Survey (2017-Mar 2020)
