# Supplementary material for: Levels of exposure markers among residents in environmentally vulnerable areas in Korea, the general population in Korea, and Asians in the United States
Source: Epidemiol Health. 2025 Feb 25;47:e2025007. doi: 10.4178/epih.e2025007 (PMC12062851; doi:10.4178/epih.e2025007)
Supplement: Supplementary Material 2. — Limit of detection and detection rate of specimens by exposure markers in the FROM study, KoNEHS IV, and NHANES [file epih-47-e2025007-Supplementary-2.docx]

**Supplementary Material** 2. Limit of detection and detection rate of specimens by exposure markers in the FROM study, KoNEHS IV, and NHANES

| Exposure markers | | Unit | FROM study | | | | KoNEHS IV (2018-2020) | | | | NHANES-Asian (2017-Mar 2020) | | | |
| --- | --- | --- | --- | --- | --- | --- | --- | --- | --- | --- | --- | --- | --- | --- |
|  |  |  | Number of specimens | Limit of detection | Detection rate (%) | Number of specimens | | Limit of detection | Detection rate (%) | Number of specimens | | Limit of detection | Detection rate (%) |  |
| Metals (Blood) | Lead | μg/dL | 1157 | 0.004-0.075 | 100.0 | 2988 | | 0.17 | 99.97 | 982 | | 0.07 | 100.0 |  |
|  | Mercury | μg/L | 1157 | 0.008-0.031 | 100.0 | 2988 | | 0.1 | 100.0 | 982 | | 0.28 | 85.4 |  |
|  | Cadmium | μg/L | 1157 | 0.019-0.085 | 100.0 | 0 | | - | - | 982 | | 0.1 | 97.9 |  |
| Metals (Urine) | Mercury | μg/L | 1157 | 0.026 | 97.3 | 4237 | | 0.04 | 98.1 | 350 | | 0.13 | 61.4 |  |
|  | Cadmium | μg/L | 1155 | 0.016-0.036 | 99.9 | 4235 | | 0.04 | 92.0 | 350 | | 0.055 | 91.4 |  |
|  | Total arsenic | μg/L | 1157 | 0.021-0.077 | 100.0 | 0 | | - | - | 350 | | 0.23 | 100.0 |  |
|  | As5+ | μg/L | 1157 | 0.027-0.190 | 48.4 | 0 | | - | - | 351 | | 0.79 | 6.6 |  |
|  | As3+ | μg/L | 1157 | 0.017-0.140 | 32.0 | 0 | | - | - | 351 | | 0.12 | 48.1 |  |
|  | Monomethylarsonic acid (MMA) | μg/L | 1157 | 0.013-0.086 | 82.3 | 0 | | - | - | 351 | | 0.2 | 58.4 |  |
| Polycyclic aromatic hydrocarbons metabolites (Urine) | 1-Hydroxypyrene | μg/L | 1157 | 0.007-0.009 | 97.8 | 4233 | | 0.044 | 69.7 | 0 | | - | - |  |
|  | 2-Naphthol | μg/L | 1157 | 0.008 | 99.8 | 4233 | | 0.033 | 99.9 | 0 | | - | - |  |
|  | 2-Hydroxyfluorene | μg/L | 1157 | 0.005-0.010 | 97.0 | 4233 | | 0.052 | 89.5 | 0 | | - | - |  |
|  | 1-Hydroxyphenanthrene | μg/L | 1157 | 0.008 | 96.5 | 4233 | | 0.037 | 63.1 | 0 | | - | - |  |
| Nicotine metabolite (Urine) | Cotinine | μg/L | 1157 | 0.194-0.418 | 92.3 | 4235 | | 0.2 | 96.6 | 0 | | - | - |  |
| Volatile organic compounds metabolites (Urine) | trans, trans-Muconic acid | μg/L | 1157 | 0.683-0.901 | 99.7 | 4228 | | 0.84 | 100.0 | 302 | | 9.81 | 83.1 |  |
|  | Benzylmercapturic acid | μg/L | 1157 | 0.350-0.439 | 98.7 | 4228 | | 0.136 | 99.5 | 0 | | - | - |  |
|  | Phenylglyoxylic acid | μg/L | 1157 | 2.312-10.644 | 99.8 | 0 | | - | - | 339 | | 12 | 99.7 |  |
|  | 2-Methylhippuric acid | μg/L | 1157 | 11.138-11.759 | 89.7 | 0 | | - | - | 339 | | 5 | 76.4 |  |
| Perfluorocarbons (Serum) | Perfluorooctanoic acid (PFOA) | μg/L | 347 | 0.050-0.051 | 99.7 | 2993 | | 0.050 | 100.0 | 0 | | - | - |  |
|  | Perfluorooctanesulfonic acid (PFOS) | μg/L | 347 | 0.049-0.056 | 100.0 | 2993 | | 0.056 | 100.0 | 0 | | - | - |  |
|  | Perfluorohexanesulfonic acid (PFHxS) | μg/L | 347 | 0.058-0.070 | 99.4 | 2993 | | 0.071 | 99.8 | 0 | | - | - |  |
|  | Perfluorononanoic acid (PFNA) | μg/L | 347 | 0.020-0.022 | 100.0 | 2993 | | 0.019 | 100.0 | 0 | | - | - |  |
|  | Perfluorodecanoic acid (PFDeA) | μg/L | 347 | 0.019-0.020 | 100.0 | 2993 | | 0.017 | 100.0 | 0 | | - | - |  |

FROM study, Forensic Research via Omics Markers in Environmental Health Vulnerable Area Study; KoNEHS IV, The Fourth Korean National Environmental Health Survey (2018-2020); NHANES, National Health and Nutrition Examination Survey (2017-Mar 2020)
