# Supplementary material for: Levels of exposure markers among residents in environmentally vulnerable areas in Korea, the general population in Korea, and Asians in the United States
Source: Epidemiol Health. 2025 Feb 25;47:e2025007. doi: 10.4178/epih.e2025007 (PMC12062851; doi:10.4178/epih.e2025007)
Supplement: Supplementary Material 3. — Distribution of exposure biomarkers among different exposure sources in the FROM study among participants older than 60 years of age [file epih-47-e2025007-Supplementary-3.docx]

**Supplementary Material** 3. Distribution of exposure biomarkers among different exposure sources in the FROM study among participants older than 60 years of age

| Exposure markers | |  | Refineries  (n=219) | Abandoned  metal mines  (n=115) | Waste  incinerator  (n=48) | Coal-fired  power plants  (n=96) | Cement  factories  (n=92) | Sprawl  (n=63) | Industrial  (n=201) |  |
| --- | --- | --- | --- | --- | --- | --- | --- | --- | --- | --- |
|  |  | Unit | Median (IQR) | Median (IQR) | Median (IQR) | Median (IQR) | Median (IQR) | Median (IQR) | Median (IQR) | p-value |
| Metals (Blood) | Lead | ㎍/dL | 2.52  (1.69-3.79) | 2.38  (1.89-2.74) | 1.48  (1.22-1.84) | 1.63  (1.28-2.10) | 1.74  (1.37-2.49) | 1.53  (1.11-1.91) | 1.41  (1.13-1.92) | <0.001 |
|  | Mercury | ㎍/L | 2.57  (1.66-3.75) | 4.71  (3.10-6.93) | 1.63  (1.25-2.52) | 4.35  (3.27-6.61) | 1.85  (1.38-2.69) | 3.50  (1.90-5.47) | 3.82  (2.57-5.20) | <0.001 |
|  | Cadmium | ㎍/L | 2.26  (1.67-3.12) | 1.73  (1.06-2.55) | 1.15  (0.87-1.58) | 1.23  (0.99-1.68) | 0.91  (0.68-1.26) | 1.39  (0.98-1.62) | 1.15  (0.92-1.55) | <0.001 |
| Metals (Urine) | Mercury | ㎍/L | 0.16  (0.09-0.31) | 0.91  (0.46-1.57) | 0.07  (0.04-0.14) | 0.21  (0.12-0.32) | 0.12  (0.06-0.20) | 0.14  (0.09-0.27) | 0.20  (0.08-0.35) | <0.001 |
|  |  | ㎍/g cr | 0.30  (0.14-0.50) | 1.11  (0.62-1.74) | 0.11  (0.04-0.17) | 0.26  (0.15-0.37) | 0.13  (0.07-0.20) | 0.33  (0.19-0.47) | 0.27  (0.18-0.48) | <0.001 |
|  | Cadmium | ㎍/L | 1.20  (0.61-2.05) | 1.51  (1.02-2.75) | 0.80  (0.41-1.33) | 0.94  (0.56-1.83) | 0.72  (0.31-1.31) | 0.52  (0.29-0.93) | 0.83  (0.38-1.41) | <0.001 |
|  |  | ㎍/g cr | 1.99  (1.21-3.10) | 1.99  (1.12-3.33) | 1.44  (0.96-2.28) | 1.10  (0.74-1.62) | 0.75  (0.48-1.15) | 1.00  (0.67-1.72) | 1.26  (0.91-1.82) | <0.001 |
|  | Total arsenic | ㎍/L | 121.91  (67.53-240.02) | 304.30  (208.80-629.10) | 106.70  (42.54-179.47) | 197.13  (115.46-369.86) | 49.57  (24.10-104.49) | 116.79  (63.83-168.62) | 110.22  (51.19-220.27) | <0.001 |
|  |  | ㎍/g cr | 210.00  (116.53-353.29) | 422.13  (239.31-733.23) | 157.60  (85.28-279.57) | 250.37  (168.35-392.54) | 52.16  (34.06-97.10) | 211.78  (123.63-364.37) | 171.28  (109.42-274.43) | <0.001 |
|  | As5+ | ㎍/L | 0.08  (0.04-0.27) | 0.47  (0.06-1.13) | 0.04  (0.04-0.15) | 0.04  (0.04-0.16) | 0.25  (0.13-0.43) | 0.02  (0.02-0.22) | 0.03  (0.03-0.22) | <0.001 |
|  |  | ㎍/g cr | 0.17  (0.06-0.44) | 0.53  (0.08-1.30) | 0.12  (0.06-0.31) | 0.07  (0.04-0.17) | 0.33  (0.19-0.52) | 0.06  (0.03-0.45) | 0.11  (0.04-0.30) | <0.001 |
|  | As3+ | ㎍/L | 0.04  (0.04-0.04) | 0.03  (0.03-0.03) | 0.04  (0.04-0.04) | 0.04  (0.04-0.23) | 1.39  (0.86-2.64) | 0.01  (0.01-0.53) | 0.03  (0.03-0.03) | <0.001 |
|  |  | ㎍/g cr | 0.08  (0.04-0.15) | 0.05  (0.04-0.15) | 0.09  (0.05-0.16) | 0.06  (0.04-0.22) | 1.68  (1.13-2.31) | 0.05  (0.03-0.79) | 0.07  (0.04-0.21) | <0.001 |
|  | Monomethylarsonic acid (MMA) | ㎍/L | 1.55  (0.71-2.95) | 1.25  (0.06-3.13) | 1.08  (0.46-1.78) | 1.31  (0.56-2.08) | 0.27  (0.10-1.15) | 23.12  (14.95-42.42) | 1.23  (0.74-2.07) | <0.001 |
|  |  | ㎍/g cr | 2.30  (1.32-3.56) | 1.57  (0.10-2.94) | 1.59  (0.93-2.77) | 1.74  (0.86-2.55) | 0.40  (0.12-0.96) | 45.43  (29.51-78.16) | 2.01  (1.26-2.85) | <0.001 |
| Polycyclic aromatic hydrocarbons metabolites (Urine) | 1-Hydroxypyrene | ㎍/L | 0.06  (0.03-0.15) | 0.10  (0.05-0.23) | 0.10  (0.04-0.20) | 0.11  (0.07-0.24) | 0.11  (0.06-0.29) | 0.13  (0.05-0.23) | 0.12  (0.06-0.24) | <0.001 |
|  |  | ㎍/g cr | 0.09  (0.05-0.19) | 0.11  (0.06-0.23) | 0.15  (0.09-0.26) | 0.17  (0.10-0.27) | 0.12  (0.08-0.26) | 0.22  (0.14-0.38) | 0.17  (0.10-0.34) | <0.001 |
|  | 2-Naphthol | ㎍/L | 2.46  (1.00-5.11) | 2.90  (0.99-8.90) | 1.87  (0.47-7.17) | 3.19  (1.51-9.06) | 2.84  (1.18-5.52) | 1.33  (0.72-4.25) | 2.01  (0.97-4.75) | 0.007 |
|  |  | ㎍/g cr | 3.67  (1.85-9.04) | 3.87  (1.53-9.47) | 2.75  (1.28-8.74) | 3.41  (1.91-8.75) | 3.34  (1.51-6.02) | 2.86  (1.50-7.51) | 3.39  (1.81-6.43) | 0.60 |
|  | 2-Hydroxyfluorene | ㎍/L | 0.11  (0.06-0.23) | 0.10  (0.05-0.20) | 0.11  (0.05-0.24) | 0.09  (0.04-0.16) | 0.07  (0.03-0.14) | 0.06  (0.03-0.14) | 0.09  (0.04-0.19) | 0.003 |
|  |  | ㎍/g cr | 0.19  (0.10-0.35) | 0.11  (0.06-0.27) | 0.17  (0.10-0.43) | 0.10  (0.05-0.18) | 0.07  (0.05-0.13) | 0.11  (0.08-0.19) | 0.13  (0.09-0.23) | <0.001 |
|  | 1-Hydroxyphenanthrene | ㎍/L | 0.08  (0.03-0.15) | 0.06  (0.03-0.11) | 0.11  (0.05-0.17) | 0.23  (0.12-0.36) | 0.14  (0.07-0.32) | 0.07  (0.03-0.13) | 0.08  (0.04-0.15) | <0.001 |
|  |  | ㎍/g cr | 0.14  (0.06-0.22) | 0.07  (0.04-0.12) | 0.14  (0.08-0.27) | 0.24  (0.17-0.34) | 0.16  (0.10-0.28) | 0.13  (0.08-0.23) | 0.15  (0.08-0.24) | <0.001 |
| Nicotine metabolite | Cotinine | ㎍/L | 3.21  (0.98-7.07) | 4.26  (1.99-8.19) | 1.07  (0.62-3.30) | 1.14  (0.55-1.82) | 1.48  (0.40-3.82) | 1.04  (0.17-2.78) | 1.05  (0.66-1.73) | <0.001 |
|  |  | ㎍/g cr | 5.18  (1.94-13.91) | 5.80  (2.46-13.52) | 1.88  (0.99-8.82) | 1.39  (0.66-2.36) | 1.43  (0.53-4.86) | 1.77  (0.31-6.31) | 1.81  (0.87-3.34) | <0.001 |
| Volatile organic compounds metabolites (Urine) | trans, trans-Muconic acid | ㎍/L | 35.54  (19.44-67.69) | 45.25  (24.07-82.47) | 36.23  (23.52-71.07) | 67.15  (33.35-117.16) | 53.98  (40.91-92.73) | 38.22  (25.79-57.30) | 55.77  (27.95-93.89) | <0.001 |
|  |  | ㎍/g cr | 57.81  (36.78-97.79) | 53.88  (32.19-83.80) | 79.98  (54.76-115.20) | 83.30  (52.49-120.67) | 65.47  (45.34-95.57) | 76.57  (55.04-117.30) | 90.72  (55.26-148.48) | <0.001 |
|  | Benzylmercapturic acid | ㎍/L | 5.31  (2.77-10.54) | 10.99  (5.12-21.65) | 2.53  (1.26-6.02) | 6.27  (3.85-11.81) | 6.74  (3.24-14.49) | 4.55  (2.34-12.64) | 5.86  (3.83-11.90) | <0.001 |
|  |  | ㎍/g cr | 8.81  (4.38-14.90) | 10.33  (5.40-24.04) | 4.70  (2.72-7.63) | 7.83  (4.84-12.54) | 7.89  (4.32-14.15) | 9.08  (5.24-23.44) | 11.26  (7.75-18.82) | <0.001 |
|  | Phenylglyoxylic acid | ㎍/L | 89.33  (39.16-169.65) | 110.13  (56.15-219.32) | 61.90  (27.83-128.65) | 219.19  (124.66-330.25) | 143.61  (97.01-248.14) | 146.50  (91.39-199.83) | 122.58  (80.67-229.68) | <0.001 |
|  |  | ㎍/g cr | 164.91  (81.22-244.06) | 161.20  (86.72-221.07) | 155.16  (51.55-231.83) | 254.15  (173.49-345.44) | 175.36  (123.00-263.80) | 302.10  (206.30-384.00) | 237.57  (156.54-318.84) | <0.001 |
|  | 2-Methylhippuric acid | ㎍/L | 45.45  (12.09-120.36) | 31.14  (7.88-94.90) | 39.24  (17.07-105.25) | 53.40  (32.84-123.60) | 88.13  (39.69-147.05) | 113.91  (65.18-179.93) | 112.01  (69.89-220.24) | <0.001 |
|  |  | ㎍/g cr | 70.39  (20.99-151.65) | 29.16  (7.88-82.42) | 55.64  (32.43-169.54) | 69.05  (44.38-122.82) | 81.26  (62.86-140.60) | 199.65  (151.09-293.75) | 201.09  (131.80-302.14) | <0.001 |

FROM study, Forensic Research via Omics Markers in Environmental Health Vulnerable Area Study

p-value estimated using Kruskal-Wallis test
