# Supplementary material for: Levels of exposure markers among residents in environmentally vulnerable areas in Korea, the general population in Korea, and Asians in the United States
Source: Epidemiol Health. 2025 Feb 25;47:e2025007. doi: 10.4178/epih.e2025007 (PMC12062851; doi:10.4178/epih.e2025007)
Supplement: Supplementary Material 4. — Distribution of exposure biomarkers by different exposure sources and control groups in the FROM study, KoNEHS IV, and NHANES-Asian among participants older than 60 years of age [file epih-47-e2025007-Supplementary-4.docx]

**Supplementary Material** 4. Distribution of exposure biomarkers by different exposure sources and control groups in the FROM study, KoNEHS IV, and NHANES-Asian among participants older than 60 years of age

| Exposure markers | |  | Refineries (n=219) | Abandoned metal mines (n=115) | Waste incinerator (n=48) | Coal-fired power plants (n=96) | Cement factories (n=92) | Sprawl (n=63) | Industrial (n=201) | Control group in the FROM study (n=204) | KoNEHS IV  (2018-2020)  (n=1566) | Asians in the NHANES (2017-Mar 2020) (n=259) | p-value |
| --- | --- | --- | --- | --- | --- | --- | --- | --- | --- | --- | --- | --- | --- |
|  |  | Unit | Median | Median | Median | Median | Median | Median | Median | Median  (IQR) | Median (IQR) | Median  (IQR) |  |
| Metals (Blood) | Lead | ㎍/dL | 2.52 | 2.38 | 1.48 | 1.63 | 1.74 | 1.53 | 1.41 | 1.27  (0.91-1.73) | 1.84  (1.38-2.37) | 1.19  (0.89-1.70) | <0.001 |
|  | Mercury | ㎍/L | 2.57 | 4.71 | 1.63 | 4.35 | 1.85 | 3.50 | 3.82 | 3.02  (2.23-4.69) | 3.13  (1.99-5.09) | 2.22  (0.88-4.19) | <0.001 |
|  | Cadmium | ㎍/L | 2.26 | 1.73 | 1.15 | 1.23 | 0.91 | 1.39 | 1.15 | 1.06  (0.76-1.48) | - | 0.53  (0.36-0.77) | <0.001 |
| Metals (Urine) | Mercury | ㎍/L | 0.16 | 0.91 | 0.07 | 0.21 | 0.12 | 0.14 | 0.20 | 0.19  (0.09-0.43) | 0.25  (0.16-0.45) | 0.22  (0.09-0.65) | <0.001 |
|  |  | ㎍/g cr | 0.30 | 1.11 | 0.11 | 0.26 | 0.13 | 0.33 | 0.27 | 0.26  (0.13-0.48) | 0.36  (0.22-0.57) | 0.41  (0.25-0.85) | <0.001 |
|  | Cadmium | ㎍/L | 1.20 | 1.51 | 0.80 | 0.94 | 0.72 | 0.52 | 0.83 | 0.91  (0.51-1.51) | 0.66  (0.38-1.14) | 0.29  (0.16-0.60) | <0.001 |
|  |  | ㎍/g cr | 1.99 | 1.99 | 1.44 | 1.10 | 0.75 | 1.00 | 1.26 | 1.09  (0.76-1.57) | 0.94  (0.60-1.39) | 0.50  (0.34-0.96) | <0.001 |
|  | Total arsenic | ㎍/L | 121.91 | 304.30 | 106.70 | 197.13 | 49.57 | 116.79 | 110.22 | 164.76  (99.41-332.10) | - | 21.25  (6.83-46.98) | <0.001 |
|  |  | ㎍/g cr | 210.00 | 422.13 | 157.60 | 250.37 | 52.16 | 211.78 | 171.28 | 210.49  (137.57-377.20) | - | 28.64  (15.70-72.47) | <0.001 |
|  | As5+ | ㎍/L | 0.08 | 0.47 | 0.04 | 0.04 | 0.25 | 0.02 | 0.03 | 0.05  (0.04-0.24) | - | 0.56  (0.56-0.56) | <0.001 |
|  |  | ㎍/g cr | 0.17 | 0.53 | 0.12 | 0.07 | 0.33 | 0.06 | 0.11 | 0.10  (0.05-0.24) | - | 0.85  (0.56-1.70) | <0.001 |
|  | As3+ | ㎍/L | 0.04 | 0.03 | 0.04 | 0.04 | 1.39 | 0.01 | 0.03 | 0.06  (0.04-1.82) | - | 0.08  (0.08-0.60) | <0.001 |
|  |  | ㎍/g cr | 0.08 | 0.05 | 0.09 | 0.06 | 1.68 | 0.05 | 0.07 | 0.17  (0.05-2.40) | - | 0.33  (0.13-0.66) | <0.001 |
|  | Monomethylarsonic acid (MMA) | ㎍/L | 1.55 | 1.25 | 1.08 | 1.31 | 0.27 | 23.12 | 1.23 | 1.45  (0.61-3.74) | - | 0.37  (0.14-0.93) | <0.001 |
|  |  | ㎍/g cr | 2.30 | 1.57 | 1.59 | 1.74 | 0.40 | 45.43 | 2.01 | 2.25  (0.80-4.47) | - | 0.67  (0.37-1.30) | <0.001 |
| Polycyclic aromatic hydrocarbons metabolites (Urine) | 1-Hydroxypyrene | ㎍/L | 0.06 | 0.10 | 0.10 | 0.11 | 0.11 | 0.13 | 0.12 | 0.13  (0.07-0.32) | 0.15  (0.03-0.30) | - | <0.001 |
|  |  | ㎍/g cr | 0.09 | 0.11 | 0.15 | 0.17 | 0.12 | 0.22 | 0.17 | 0.17  (0.09-0.35) | 0.17  (0.07-0.40) | - | <0.001 |
|  | 2-Naphthol | ㎍/L | 2.46 | 2.90 | 1.87 | 3.19 | 2.84 | 1.33 | 2.01 | 2.17  (1.01-4.88) | 2.17  (1.03-5.53) | - | 0.01 |
|  |  | ㎍/g cr | 3.67 | 3.87 | 2.75 | 3.41 | 3.34 | 2.86 | 3.39 | 2.49  (1.25-6.27) | 2.82  (1.60-6.47) | - | 0.02 |
|  | 2-Hydroxyfluorene | ㎍/L | 0.11 | 0.10 | 0.11 | 0.09 | 0.07 | 0.06 | 0.09 | 0.08  (0.03-0.15) | 0.24  (0.11-0.47) | - | <0.001 |
|  |  | ㎍/g cr | 0.19 | 0.11 | 0.17 | 0.10 | 0.07 | 0.11 | 0.13 | 0.09  (0.05-0.18) | 0.35  (0.17-0.59) | - | <0.001 |
|  | 1-Hydroxyphenanthrene | ㎍/L | 0.08 | 0.06 | 0.11 | 0.23 | 0.14 | 0.07 | 0.08 | 0.08  (0.04-0.17) | 0.10  (0.03-0.21) | - | <0.001 |
|  |  | ㎍/g cr | 0.14 | 0.07 | 0.14 | 0.24 | 0.16 | 0.13 | 0.15 | 0.10  (0.06-0.20) | 0.12  (0.04-0.30) | - | <0.001 |
| Nicotine metabolite | Cotinine | ㎍/L | 3.21 | 4.26 | 1.07 | 1.14 | 1.48 | 1.04 | 1.05 | 2.02  (0.90-5.58) | 2.00  (0.84-5.80) | - | <0.001 |
|  |  | ㎍/g cr | 5.18 | 5.80 | 1.88 | 1.39 | 1.43 | 1.77 | 1.81 | 2.79  (1.22-8.07) | 2.32  (1.19-7.16) | - | <0.001 |
| Volatile organic compounds metabolites (Urine) | trans, trans-Muconic acid | ㎍/L | 35.54 | 45.25 | 36.23 | 67.15 | 53.98 | 38.22 | 55.77 | 53.57  (28.65-99.26) | 43.68  (26.07-82.24) | 20.50  (11.05-38.65) | <0.001 |
|  |  | ㎍/g cr | 57.81 | 53.88 | 79.98 | 83.30 | 65.47 | 76.57 | 90.72 | 63.63  (40.17-107.04) | 59.39  (37.00-100.02) | 36.00  (24.16-67.84) | <0.001 |
|  | Benzylmercapturic acid | ㎍/L | 5.31 | 10.99 | 2.53 | 6.27 | 6.74 | 4.55 | 5.86 | 9.00  (4.17-18.19) | 5.15  (2.75-10.67) | - | <0.001 |
|  |  | ㎍/g cr | 8.81 | 10.33 | 4.70 | 7.83 | 7.89 | 9.08 | 11.26 | 10.92  (5.48-23.95) | 6.75  (4.16-12.18) | - | <0.001 |
|  | Phenylglyoxylic acid | ㎍/L | 89.33 | 110.13 | 61.90 | 219.19 | 143.61 | 146.50 | 122.58 | 194.40  (110.55-335.30) | - | 161.00  (77.12-226.75) | <0.001 |
|  |  | ㎍/g cr | 164.91 | 161.20 | 155.16 | 254.15 | 175.36 | 302.10 | 237.57 | 255.61  (163.56-388.09) | - | 244.46  (187.27-321.40) | <0.001 |
|  | 2-Methylhippuric acid | ㎍/L | 45.45 | 31.14 | 39.24 | 53.40 | 88.13 | 113.91 | 112.01 | 106.33  (54.23-309.55) | - | 10.50  (3.54-24.75) | <0.001 |
|  |  | ㎍/g cr | 70.39 | 29.16 | 55.64 | 69.05 | 81.26 | 199.65 | 201.09 | 121.73  (72.40-321.60) | - | 17.14  (10.15-45.59) | <0.001 |

FROM study, Forensic Research via Omics Markers in Environmental Health Vulnerable Area Study; KoNEHS IV, The Fourth Korean National Environmental Health Survey (2018-2020); NHANES, National Health and Nutrition Examination Survey (2017-Mar 2020)

p-value estimated using Kruskal-Wallis test
